# Is Childhood Obesity Associated with Bone Density and Strength in Adulthood?

**DOI:** 10.4061/2010/904806

**Published:** 2010-06-20

**Authors:** Kirsti Uusi-Rasi, Pekka Kannus, Matti Pasanen, Harri Sievänen

**Affiliations:** ^1^The UKK Institute for Health Promotion Research, Bone Research Group, Tampere, P.O. Box 30, 33501 Tampere, Finland; ^2^Research Department, University Hospital, Tampere, P.O. Box 2000, 33521 Tampere, Finland; ^3^Medical School, University of Tampere, 33014 University of Tampere, Finland; ^4^Division of Orthopaedics and Traumatology, Department of Trauma, Musculoskeletal Surgery and Rehabilitation, Tampere University Hospital, P.O. Box 2000, 33521 Tampere, Finland

## Abstract

Associations between childhood obesity and adult bone traits were assessed among 62 obese premenopausal women, of which 12 had been obese since childhood (ObC), and 50 had gained excess weight in adulthood (ObA). Body composition and bone mineral content (BMC) of the total body, spine, and proximal femur were assessed with DXA. Total cross-sectional area and cortical (diaphyseal CoD) and trabecular (epiphyseal TrD) bone density of the radius and tibia were measured with pQCT. Compared to ObA-group, ObC-group was 5.2 cm taller having 2.5 and 3.5 kg more lean and fat mass, respectively. Depending on the statistical adjustment, ObC-group had 5–10% greater TrD both in tibia and in radius. The remaining bone traits did not significantly differ between the groups. Current preliminary observations bring up an interesting question whether childhood obesity can result in denser trabecular bone in adulthood. However, prudence must be exercised in the statistical adjustment.

## 1. Introduction

Compared to normal-weight adults, overweight and obese persons have not only greater bone mineral density (BMD), but they lose bone at a slower pace, and may even have a reduced risk of fragility fractures [1, 2]. As regards the effect of childhood obesity on bone mass, findings are inconsistent [3–5]. Some studies have attributed greater bone mass to excess body weight in the growing years [5–8] but it has also been suggested that obese children have lower bone mass for a given weight [9–11]. 

In principle, the greater bone mass in obese subjects could simply be a consequence of increased body mass. While the weight load per se can stimulate bone formation, obese subjects also have moderate increase in muscle mass [12, 13], which is an important determinant of bone mass and strength [14]. In absolute terms bone mass and strength have been shown to be greater in obese women but reduced relative to body weight varying in proportion to lean mass, not to body weight or fat mass [15]. Interestingly, some studies have suggested that fat mass, the other major component of body weight, may also stimulate bone accrual in growing children, but these results have remained inconsistent showing both positive [16, 17] and negative associations [11, 13, 18]. 

Despite the apparent simplicity, the relationship between body weight and bone mass and strength is not straightforward. While obesity is represented by increased body weight, and thus the mass needed to move during habitual activities, the incident muscular contractions, particularly the magnitude of force, the rate of force production, and the total amount of contractions play a more important role than body weight. Body weight alone imposes relatively small and static load on bones (corresponding to Earth's gravity, G), whereas the load can be substantially amplified in different activities by muscle actions [19]. Theoretically, a physically active obese individual has stronger muscles than a sedentary, similarly obese person. Consequently, the greater muscle force would permit more bone remodeling and thus result in increased bone mass and strength in the former. On the other hand, if an obese individual is sedentary or becomes inactive and loses muscle mass, incident muscle forces imposed on bones decline and osteopenia could ensue no matter how obese the individual might be [12]. However, it is not yet known whether the influence of increased body weight on bones would be stronger in childhood and persist until adulthood.

The conventional paradigm suggests that obesity results in greater bone mass as a consequence of greater body weight and could thus protect against osteoporosis. In this cross-sectional study we hypothesized that those women who have been obese since childhood would show some benefit in bone traits compared to women who have gained extra weight not earlier than in adulthood. This hypothesis was based on observations that the period of adolescent growth provides the window of opportunity for efficient bone accrual [20]. In addition, we evaluated whether statistical adjusting for different anthropometric variables affects the results, and if so, to what extent.

## 2. Methods

### 2.1. Subjects

Sixty-two obese (BMI >30) clinically healthy premenopausal women with the age range of 25 to 45 years participated in the study [21]. According to self-reports, 12 of these women had been obese since childhood, and 50 had gained excess weight after maturity. None of the subjects had evidence for any disease, prior injuries, or drug use (hormonal contraceptives were allowed) that would have affected their skeleton. The study protocol was approved by the Ethics Committee of The Pirkanmaa Hospital District, Tampere, Finland, and each participant gave her written informed consent.

### 2.2. Health History Questionnaire

Information on self-reported health, injuries, medication, diseases, diet, nonpregnant weight at the age of 25, history of weight cycling, weight loss attempts, menstrual status, and lifestyle factors such as current physical activity, smoking, and consumption of alcohol was obtained with a questionnaire. The questionnaire was completed in an interview.

### 2.3. Measurements

**Physical Activity**

History of physical activity was assessed in an interview, and current daily walking steps were measured with a pedometer (Omron HL-112-E, Omron Healthcare Europe) on six days.

**Muscle Performance**

The maximal isometric leg extension force (N/kg) was measured by a strain gauge dynamometer at a knee angle of 110 degrees (Tamtron, Tampere, Finland). The maximal take-off force (N/kg) and power (W/kg) during a vertical counter-movement jump were measured on a force-plate (Kistler Ergojump 1.04, Kistler Instrumente AG, Winterthur, Switzerland). Functional agility (s) was evaluated by a figure-8 running test and the grip strength of both forearms (kg) with a standard grip strength meter.

**Anthropometry and Body Composition**

Body height was measured to the nearest 0.1 cm and body weight to the nearest 0.1 kg with a high-precision scale with the participants wearing only their underwear. Waist circumference was measured midway between the lowest rib and the iliac crest and hip circumference at the tip of the greater trochanter. Body composition (fat mass and lean mass) and the android (trunk) and gynoid (hip) fat-% were assessed with dual-energy X-ray absorptiometry (DXA, Lunar Prodigy Advance, GE Lunar, Madison, WI, USA). In our laboratory, the *in vivo* precision (coefficient variation, CV%) based on repeated scans of 27 subjects with repositioning is 1.3% for the fat mass and 0.8% for the lean mass (Sievänen, unpublished data).

**Bone Mass and Structure**

Bone mineral content (BMC, g) of the total body, BMC, and bone mineral density (BMD, g/cm^2^) of the left proximal femur and lumbar spine were assessed with DXA (Lunar Prodigy Advance). Femoral neck structure was assessed using the Advanced Hip Analysis (AHA) which provided data on cross-sectional area occupied by bone mineral (CSA, mm^2^, an index of bone strength against compression), section modulus (Z, mm^3^, an index of bone strength against bending), and outer diameter (Width, mm). In our laboratory, the *in vivo* precision is 1.4% for the total body BMC, about 1% for the lumbar spine BMC, 1.5% for the femoral neck BMC, and about 3% for the trochanter BMC (Sievänen 2005, unpublished data). The *in vivo* precision for CSA, Z, and Width is 2.3%, 3.8%, and 1.2%, respectively.

In addition to the DXA measurements, the left radius and tibia were measured with pQCT (Norland/Stratec XCT 3000, Pforzheim, Germany). The tomographic slices were taken from the shaft and distal part of the tibia (50% and 5% from the distal endplate of the tibia, resp.) and of the radius (30% and 4% from the distal endplate of the radius, resp.) according to our standard procedures [22]. For the shaft regions, the analyzed bone traits were total cross-sectional area (ToA, mm^2^), cortical density (CoD, mg/cm^3^), and density-weighted polar section modulus (SSI, mm^3^, an index of bone strength against bending). For the distal parts of the radius and tibia the traits were ToA, trabecular density (TrD, mg/cm^3^), and SSI. In our laboratory, the *in vivo* precision of the pQCT traits is between 0.7% (CoD of the tibial shaft) and 7.7% (SSI of the distal radius) [22].

**Statistical Analyses**

Mean and standard deviations (SD) were used as descriptive statistics. Between-group differences were evaluated by an analysis of covariance, and as possible confounding factors body height, fat, and lean mass were used as covariates. Since the body mass index (BMI) is a commonly used covariate in the pertinent literature, the analyses were adjusted for BMI for comparison. Linear regression analyses were used to find the predictors for bone traits; body height, lean mass, fat mass, and childhood obesity were included as the dichotomous variable (ObC = 0; ObA = 1) in the regression models.

## 3. Results

The group characteristics (mean, SD) are given in Table 1. The ObC-group was on average 5.2 cm taller and compared to the ObA-group they had 2.5 kg and 3.5 kg more lean and fat mass, respectively. However, difference in mean relative body fat and lean mass was small indicating similar body composition in both groups for given body height in adulthood. As shown in Table 1, the muscle strength, functional agility, and steps/day were similar between the two groups. 

The DXA-based bone mass and strength were greater in the ObC-group than that those in the ObA-group (Table 2). Adjusting for BMI had practically no effect on the mean differences, whereas the adjustment for height, fat, and lean mass affected the results, which were no more statistically significant. 

Although the ObC-group tended to have greater TrD both at the distal radius and at the distal tibia, cortical density was similar in both groups. There was also a tendency for greater total bone cross-sectional area in ObC-group especially in the weight-bearing tibia. However, this difference disappeared after adjusting for body height and fat and lean tissue. In the nonweight-bearing radius, cross-sectional bone area was similar in both groups, and adjusting for anthropometric variables did not affect the results.

The most significant predictors for bone traits were height and lean mass (Tables 3, 4, and 5). Height was positively associated with bone mass and strength at the axial skeleton, while lean mass was the strongest correlate for most bone traits at the appendicular skeleton.

## 4. Discussion

According to our findings, women who had been obese since childhood were taller than women who had gained weight in adulthood. Obese children tend to be tall for their age and advanced in sexual and skeletal development, but not necessarily in terms of adult height, which was found in the present study. Since body size is strongly associated with bone mass and strength, and taller people have more massive skeleton than their smaller counterparts, body height and weight, or their aggregate measure BMI, are commonly used in literature as covariates to statistically control for the between-group differences in anthropometric variables. Besides the conventional variables, we measured both fat and lean tissue (the main components of body weight) in this study, and both of these variables were used as covariates instead of pure body weight. According to our findings, body height is a much stronger predictor of bone mass and strength than body weight, while lean mass turned out to be a stronger predictor for pQCT-assessed appendicular bone traits than height.

Taller persons usually have bigger bone cross-sections, and in line with this notion the total CSA of the weight-bearing tibia was greater in the ObC group than in the ObA group. In this respect, it is recalled that BMI (by definition = weight /height^2^ in meters) does not totally correct differences in body size, which was also shown by our results. Thus we argue that BMI is not an appropriate variable in adjusting for different body sizes. In general, we found that BMI-adjusted DXA- and pQCT traits were similar to unadjusted crude differences. However, after adjusting for body height, lean, and fat mass the mean differences declined and were no statistically significant. With regard to bone traits which are basically size-independent, such as cortical density, the adjustment did not affect the results. To sum up, if it is not possible to separate lean, and fat mass from each other, use of total body weight, in addition to height, better takes into account the differences in body size than BMI, and the former approach is recommended. 

Increased biomechanical loading of the skeleton due to increased body weight and increased lean mass (~muscle forces) may contribute to greater bone mass and size. Obese persons (both children and adults) have to move greater body mass in habitual physical activity compared to the persons with normal body weight, which implies greater loading on bones provided that the intensity and the amount of exercise are similar. Some previous DXA-based studies, but not all, have shown that obese children have normal or increased bone mass relative to healthy weight peers [4, 6]. In contrast, Goulding et al. found decreased bone mass in obese children relative to bone size and body weight [9]. Also a Canadian cohort showed that the greater bone strength in obese children was appropriate for their lean mass, not their fat mass [8]. In a recently published pQCT-study the obese children had similar cortical density but greater radial and trabecular density than normal weight children [7], which gives support to our finding. Some previous studies have also suggested that fat mass may modulate periosteal bone growth in childhood [16, 23], but we did not find such associations in adulthood, the weak correlation with the bone strength index at the distal radius excluded.

Due to cross-sectional study design, we cannot definitely say whether the ObC and ObA groups differed essentially in their habits of physical activity in childhood (commuting to school, playing, and related activities). However, at the time of the study the participants were 40 years old on average, which means that when they were school kids in 1970s, a decade when children in Finland commonly walked or cycled to school, played outdoor games, spent less time watching TV, and had no internet games or computers. This being the apparent case, their bones received versatile dynamic loading in childhood and thus an adequate mechanical stimulus. In contrast, it is well possible that when the present-day children are grown-up, the situation is different. Nowadays children are more and more transported to school by car or bus, and instead of playing outdoor games, they are engaged in computer games or internet in their leisure activities. The obesity epidemic is associated with sedentary life style, which means substantially less dynamic loading on bones and most likely more fragile bone in adulthood. 

Similarly we can only speculate about hormonal effects on bone development in childhood and adolescence. However, extra weight in the form of fat mass, besides its potential mechanical influence on bones [12, 14], may increase production of many hormones, such as estrogen, leptin, and insulin [13, 24]. Adipose tissue is known to express aromatase enzymes that convert steroid precursors to estrogen, which has been reported to stimulate and suppress periosteal bone growth in childhood [25]. Obese children may enter puberty earlier than normal-weight children, and they also achieve similar estradiol levels and bone ages at significantly earlier chronological ages than nonobese children [26, 27]. Hormonal actions may thereby potentially contribute to the increased linear growth and skeletal mass observed in childhood obesity [28]. Further studies are, however, needed to elucidate effects of adipose tissue and adipose-modulated hormones on adult bone mass and strength. 

The strength of this study was the use of pQCT besides the conventional DXA, which allowed not only the differentiation of trabecular and cortical bone but also information on bone geometry. In addition, bone traits both at the weight-bearing tibia and nonweight-bearing radius were evaluated, and the muscle performance of the subjects was assessed. This study also had limitations the most important being the small sample size. Only twelve women reported that they had been obese since childhood. Obviously this rendered the study underpowered to statistically uncover meaningful differences, such as the properly adjusted 5 to 8% differences in trabecular density. Second, our study group represented a convenience sample and focused only on premenopausal women. Third, the analyses were based on a self-reported retrospective data, and we do not have exact body weight data from childhood over the adolescent period. Fourth, the ObC group was about 5 cm taller, which may be just a coincidence. Childhood obesity has been shown to be associated with greater height-for-age, advanced maturation for age, and greater lean mass for height, although the advantage in growth gradually decreases and probably does not account for taller height in adulthood [4]. It is, however, recalled that the difference in body height was statistically controlled for and it did not affect the present conclusions.

In conclusion, childhood obesity seemed to be slightly associated with denser trabecular bone in adulthood in both weight-bearing and nonweight-bearing bones among obese premenopausal women, but not with cortical density, neither was childhood obesity associated with total cross-sectional bone area. It is recalled that the study sample was small and the between-group mean differences did not reach statistical significance. Nevertheless, the results bring up an interesting question, whether childhood obesity can result in denser trabecular bone in adulthood. Finally, in this kind of research attention must be paid on proper adjustment of the data.

## Figures and Tables

**Table 1:** Group characteristics (SD) among women who have been obese since childhood (ObC) and women who have become obese in adulthood.

	ObC *n* = 12	ObA *n* = 50	*P*
Height, cm	170.0 (8.2)	164.8 (5.6)	.053
Weight, kg	98.2 (16.6)	92.1 (14.0)	.25
Weight at the age of 25, kg	81.9 (14.9)	69.5 (8.6)	.016
BMI	33.8 (3.4)	33.9 (4.8)	.93
Age, yrs	37.8 (5.9)	40.5 (4.8)	.17
Fat mass, kg	45.4 (10.0)	41.9 (9.5)	.29
Lean mass, kg	49.1 (8.4)	46.6 (5.5)	.35
Fat, %	47.9 (4.5)	47.0 (4.4)	.53
Waist circumference, cm	109 (15)	107 (13)	.75
Hip circumference, cm	119 (9)	117 (10)	.55
Android fat, %	53.1 (6.2)	53.0 (5.0)	.94
Gynoid fat, %	51.0 (5.4)	51.0 (5.6)	1.00
Grip strength, kg	37.4 (5.2)	35.7 (4.4)	.31
Time of figure-8 run, s	15.6 (1.6)	15.6 (1.4)	.92
Isometric leg extension, N/kg	25.8 (6.9)	25.9 (8.2)	.94
Jumping force, N/kg	20.2 (2.9)	21.5 (3.3)	.22
Jumping power, W/kg	31.7 (5.6)	31.5 (5.5)	.95
Steps/day	8590 (3486)	7687 (2939)	.42

**Table 2:** Unadjusted bone values (SD), BMI-adjusted, and height, fat, and lean mass-adjusted between-group (ObC versus ObA) mean differences (95% CI).

	ObC, *n* = 12	ObA, *n* = 50	unadjusted mean difference, %	BMI adjusted mean difference, %	height, fat and lean adjusted mean difference, %
Total body BMC, g	3233 (536)	2904 (386)	8.4 (0.1 to 17.4)	11.0 (1.4 to 21.5)	3.4 (−4.6 to 12.2)
Lumbar spine BMC	80.12 (17.57)	69.30 (12.10)	14.8 (2.3 to 28.9)	14.8 (2.2 to 29.0)	6.2 (−4.8 to 18.4)
Femoral neck BMC	5.72 (0.93)	5.09 (0.76)	12.1 (1.8 to 23.3)	12.1 (1.9 to 23.4)	6.0 (−3.2 to 16.1)
Trochanter BMC	12.34 (2.84)	11.43 (2.57)	7.8 (−6.4 to 24.2)	7.9 (−6.4 to 24.3)	−0.7 (−13.3 to 13.9)
Femoral neck Z, mm^3^	776 (176)	660 (113)	16.7 (4.2 to 30.6)	16.7 (4.2 to 30.7)	8.6 (−2.0 to 20.4)

Distal radius					
TrD, mg/cm^3^	224.1 (35.5)	203.9 (31.1)	9.8 (−0.9 to 21.7)	9.8 (−1.0 to 21.9)	7.9 (−3.4 to 20.4)
ToA, mm^2^	277.5 (41.0)	286.0 (44.7)	−2.8 (−12.5 to 8.0)	3.0 (−10.4 to 18.4)	−5.8 (−14.7 to 4.1)
SSI, mm^3^	376.9 (86.0)	365.1 (74.0)	2.9 (−10.4 to 18.1)	3.0 (−10.4 to 18.4)	4.0 (−9.5 to 19.5)

Radial shaft					
CoD, mg/cm^3^	1201.6 (15.7)	1202.4 (19.5)	−0.1 (−1.1 to 1.0)	−0.1 (−1.1 to 1.0)	0.0 (−1.1 to 1.2)
ToA, mm^2^	105.9 (12.0)	105.8 (14.3)	0.5 (−8.0 to 9.8)	0.7 (−7.7 to 10.0)	−2.2 (−10.2 to 6.6)
SSI, mm^3^	215.1 (32.3)	215.8 (43.1)	0.5 (−11.5 to 14.2)	0.8 (−11.2 to 14.5)	−3.5 (−15.0 to 9.5)

Distal tibia					
TrD, mg/cm^3^	254.0 (27.0)	238.0 (27.6)	6.8 (−0.7 to 15.0)	6.9 (−0.7 to 15.0)	5.4 (−2.5 to 13.9)
ToA, mm^2^	897.6 (112.3)	848.4 (105.4)	5.8 (−2.4 to 14.7)	5.9 (−2.1 to 14.6)	0.5 (−5.8 to 7.1)
SSI, mm^3^	1523.9 (385.7)	1402.2 (288.7)	7.5 (−6.6 to 23.7)	7.6 (−6.3 to 23.6)	2.4 (−11.2 to 18.0)

Tibial shaft					
CoD, mg/cm^3^	1145.5 (13.0)	1143.8 (22.1)	0.2 (−1.0 to 1.3)	0.2 (−1.0 to 1.3)	0.3 (−0.9 to 1.6)
ToA, mm^2^	501.5 (41.3)	471.0 (53.3)	6.8 (−0.4 to 14.5)	6.9 (0.0 to 14.3)	2.6 (−3.4 to 9.0)
SSI, mm^3^	2138.3 (213.6)	1931.1 (331.0)	11.8 (0.8 to 24.0)	11.9 (1.3 to 23.8)	5.9 (−3.9 to 16.7)

**Table 3:** Multiple linear regressions showing the simultaneous effects of height, lean, and fat mass and age of weight gain on bone traits in the axial skeleton.

Axial skeleton	*β*	SE	*P*
Total body BMC, g			
Height, cm	40.7	9.4	<.001
Fat mass, g	6.7	6.0	.27
Lean mass, g	−11.6	11.1	.30
Age of weight gain, (ObC = 0; ObA = 1)	−119.8	121.3	.33

Femoral neck BMC, g			
Height, cm	0.046	0.019	.019
Fat mass, g	−0.001	0.012	.97
Lean mass, g	0.026	0.022	.25
Age of weight gain, (ObC = 0; ObA = 1)	−0.322	0.243	.19

Trochanter BMC, g			
Height, cm	0.151	0.063	.021
Fat mass, g	0.003	0.040	.95
Lean mass, g	0.063	0.074	.40
Age of weight gain, (ObC = 0; ObA = 1)	0.043	0.816	.96

Lumbar spine BMC, g			
Height, cm	1.15	0.31	.001
Fat mass, g	0.042	0.198	.83
Lean mass, g	−0.14	0.37	.71
Age of weight gain, (ObC = 0; ObA = 1)	−4.97	4.02	.22

Femoral neck Z, mm^3^			
Height, cm	8.70	2.88	.004
Fat mass, g	−1.64	1.83	.38
Lean mass, g	4.94	3.39	.15
Age of weight gain, (ObC = 0; ObA = 1)	−63.77	37.17	.092

**Table 4:** Multiple linear regressions showing the simultaneous effects of height, lean, and fat mass and age of weight gain on bone traits in the appendicular nonweight-bearing skeleton.

Appendicular nonweight-bearing skeleton	*β*	SE	*P*
Distal radius TrD, mg/cm^3^			
Height, cm	0.64	0.86	.46
Fat mass, g	0.44	0.55	.42
Lean mass, g	−0.73	1.06	.5
Age of weight gain, (ObC = 0; ObA = 1)	−16.88	11.37	.14

Distal radius SSI, mm^3^			
Height, cm	−0.7	1.94	.72
Fat mass, g	−2.25	1.25	.076
Lean mass, g	6.18	2.41	.013
Age of weight gain, (ObC = 0; ObA = 1)	−14.75	25.7	.57

Distal radius ToA, mm^2^			
Height, cm	1.57	1.05	.14
Fat mass, g	−0.58	0.67	.39
Lean mass, g	2.96	1.3	.026
Age of weight gain, (ObC = 0; ObA = 1)	17.12	13.87	.22

Radial shaft CoD, mg/cm^3^			
Height, cm	0.079	0.5	.88
Fat mass, g	−0.106	0.32	.74
Lean mass, g	−0.77	0.62	.22
Age of weight gain, (ObC = 0; ObA = 1)	0.03	6.58	.996

Radial shaft SSI, mm^3^			
Height, cm	1.6	1.04	.13
Fat mass, g	0.29	0.67	.67
Lean mass, g	1.12	1.29	.39
Age of weight gain, (ObC = 0; ObA = 1)	10.01	13.78	.47

Radial shaft ToA, mm^2^			
Height, cm	0.42	0.34	.17
Fat mass, g	0.01	0.22	.85
Lean mass, g	0.65	0.42	.13
Age of weight gain, (ObC = 0; ObA = 1)	2.79	4.51	.54

**Table 5:** Multiple linear regressions showing the simultaneous effects of height, lean, and fat mass and age of weight gain on bone traits in the appendicular weight-bearing skeleton.

Appendicular weight-bearing skeleton	*β*	SE	*P*
Distal tibia TrD, mg/cm^3^			
Height, cm	0.52	0.73	0.47
Fat mass, g	0.76	0.46	0.1
Lean mass, g	−0.89	0.86	0.3
Age of weight gain, (ObC = 0; ObA = 1)	−12.76	9.39	0.18

Distal tibia SSI, mm^3^			
Height, cm	6.04	7.75	0.44
Fat mass, g	0.56	4.93	0.91
Lean mass, g	14.49	9.11	0.12
Age of weight gain, (ObC = 0; ObA = 1)	−52.17	99.95	0.6

Distal tibia ToA mm^2^,			
Height, cm	5.24	2.12	0.016
Fat mass, g	−1.64	1.35	0.23
Lean mass, g	9.26	2.49	<.001
Age of weight gain, (ObC = 0; ObA = 1)	−4.43	27.31	0.87

Tibial shaft CoD, mg/cm^3^			
Height, cm	0.026	0.549	0.96
Fat mass, g	0.11	0.35	0.76
Lean mass, g	−0.87	0.65	0.19
Age of weight gain, (ObC = 0; ObA = 1)	−3.38	7.08	0.64

Tibial shaft SSI, mm^3^			
Height, cm	10.47	7.23	0.15
Fat mass, g	2.77	4.6	0.55
Lean mass, g	16.4	8.5	0.059
Time of weight gain, (ObC = 0; ObA = 1)	−102.09	93.25	0.28

Tibial shaft ToA, mm^2^			
Height, cm	1.75	1.09	0.11
Fat mass, g	0.06	0.69	0.93
Lean mass, g	3.93	1.28	0.003
Age of weight gain, (ObC=0; ObA=1)	−11.33	14.02	0.42
